# ER stress and UPR in Alzheimer’s disease: mechanisms, pathogenesis, treatments

**DOI:** 10.1038/s41419-022-05153-5

**Published:** 2022-08-15

**Authors:** Amir Ajoolabady, Dan Lindholm, Jun Ren, Domenico Pratico

**Affiliations:** 1 Shanghai Institute of Cardiovascular Diseases, Department of Cardiology, Zhongshan Hospital Fudan University, Shanghai, China; 2 Minerva Foundation Institute for Medical Research, Helsinki, Finland; 3 Department of Biochemistry and Developmental Biology, Faculty of Medicine, University of Helsinki, Helsinki, Finland; 4 Department of Laboratory Medicine and Pathology, University of Washington Seattle, Seattle, WA 98195 USA; 5 Alzheimer’s Center at Temple, Lewis Katz School of Medicine, Temple University, Philadelphia, PA 19140 USA

**Keywords:** Neuroscience, Pathogenesis

## Abstract

Alzheimer’s disease (AD) is a devastating neurodegenerative disorder characterized by gradual loss of memory and cognitive function, which constitutes a heavy burden on the healthcare system globally. Current therapeutics to interfere with the underlying disease process in AD is still under development. Although many efforts have centered on the toxic forms of Aβ to effectively tackle AD, considering the unsatisfactory results so far it is vital to examine other targets and therapeutic approaches as well. The endoplasmic reticulum (ER) stress refers to the build-up of unfolded or misfolded proteins within the ER, thus, perturbing the ER and cellular homeostasis. Emerging evidence indicates that ER stress contributes to the onset and development of AD. A thorough elucidation of ER stress machinery in AD pathology may help to open up new therapeutic avenues in the management of this devastating condition to relieve the cognitive dementia symptoms. Herein, we aim at deciphering the unique role of ER stress in AD pathogenesis, reviewing key findings, and existing controversy in an attempt to summarize plausible therapeutic interventions in the management of AD pathophysiology.

## Facts

- As Aβ and tau deposition induces ER stress, adaptive UPR signaling is activated to reverse ER stress and regain the ER homeostasis thereby preventing exacerbation of AD pathogenesis, suggesting the neurons’ potential to counter mild/basic ER stress.
- Advanced stages of AD pathology are associated with irreversible ER stress and excessive/maladaptive UPR activation, leading to neuroinflammation and or neuronal cell death.
- While AD-associated adaptive and maladaptive UPR signalings have shared components, the severity of ER stress or UPR activation is believed to differentiate between the adaptive and maladaptive responses.
- Other than major UPR components, the secondary effectors of maladaptive UPR (e.g., PERK, IRE1, ATF6) play a role in the differentiation between adaptive and maladaptive responses in neurons upon AD onset/progression.

## Open question

- To what extent ER stress is reversible in neurons via adaptive UPR signaling ensuing Aβ and or tau deposition?
- Besides the excessivity/severity of ER stress and UPR activation, which other factors determine between adaptive and maladaptive UPR signaling in either the protection or death of neurons in AD?
- To what extent targeting components of maladaptive UPR could be effective in the alleviation of AD pathophysiology? Which compounds are suitable for this purpose and which ones will meet the clinical goals?

## Introduction: unfolded/misfolded proteins in Alzheimer’s disease etiology

Neurons are polarized cells with functionally and structurally distinct compartments encompassing axons, dendrites, and soma [1]. Axons denote the long portion of neurons that protrudes from the soma and extends into axon terminals [2]. Dendrites are appendages much shorter than axons, forming highly branched and elaborated networks for communications between cells [3]. Neurons are highly dependent on oxidative metabolism for their functions and for the transmission and processing of information, exposing them to the burden of enhanced cell stress [4]. As such, neurons are prone to stressful environments, and the accumulation of damaged/misfolded proteins due to the inability of neurons to undergo *mitosis*, a process that abates protein accumulation in mitotic cells [5]. Furthermore, most organisms are unable to regenerate neurons due to the terminally differentiated nature of these cells. To this end, neurons typically adopt robust responses to stressors evoked by accumulated misfolded proteins, particularly, in the setting of pathological conditions such as neurodegenerative diseases [6]. Damaged, misfolded, and unfolded proteins contribute to the storage and conformational anomalies within the ER [7, 8], triggering the onset of ER stress, a process commonly seen in various neurodegenerative disorders including Alzheimer’s disease (AD) and Parkinson’s disease (PD) [9, 10]. The physiological folding, oligomerization, and posttranslational modification of proteins occur in the endoplasmic reticulum (ER) and it’s important for maintaining the functionality and survival of the cell [11–13]. Dysfunctions in these processes can lead to the formation and assembly of misfolded proteins in the cell, provoking pathological insults [14]. Protein misfolding is the essential event in the pathogenesis of many neurodegenerative disorders [15]. Various neurodegenerative diseases display specific types of misfolded proteins [16]. For example, AD, PD, Huntington’s disease, and amyotrophic lateral sclerosis (ALS) are featured by a clinically silent period characterized by progressive aggregation and accumulation of aberrant proteins in the brain, resulting in altered function of synapses and ultimately, neurodegeneration [17]. Hence, these conditions are also named “protein misfolding diseases”, affecting the peripheral/central nervous system [18].

As the most prevalent type of dementia, AD afflicts over 25 million people globally, particularly, the elderly aged above 85 years old. Currently, no effective treatment is available to retard the onset and progression of AD [19]. Pathologically, AD displays the intracellular accumulation of phosphorylated tau protein and extracellular aggregation of amyloid β (Aβ) peptides in the brain, leading to a progressive albeit gradual impairment in cognitive function, ultimately, clinical *dementia* [16, 20]. Histopathologically, the major characteristic of AD is neurofibrillary tangles (NFT) formed by hyperphosphorylated tau proteins and amyloid plaques formed by insoluble Aβ peptides [21, 22]. Moreover, the progressive cerebral buildup of these aberrant proteins triggers neuroinflammation, with activation of glial cells, and ultimately neurodegeneration [23]. Overall, AD is featured by excessive production, oligomerization, and deposition of Aβ, as well as the buildup of hyperphosphorylated tau proteins, forming NFT [24–26]. The amyloid-beta precursor protein (APP) undergoes a sequential cleavage governed by BACE1 (aka β-secretase) and γ-secretase protein complex, which is composed of PSEN1, PSEN2, NCSTN, APH1A, and PSENEN proteins, to yield Aβ40 and Aβ42 peptides [27, 28]. These peptides will form diffusible/soluble oligomers and fibrils or insoluble plaques in the extracellular environment, all of which are to a varying degree toxic to the neurons [29]. Under physiological conditions, tau protein mediates the stabilization of microtubules in healthy neurons, where its phosphorylation status is kept low by a delicate balance between kinases and phosphatases. However, for unknown mechanisms tau can become highly phosphorylated and because of that lose the affinity for the microtubules, forming fibrils that tend to aggregate and accumulate in neuronal cytoplasm as NFT [30]. Genetic mutations involving *PSEN1*, *PSEN2*, and *APP* genes, result in an increased production of aggregatable subtype of Aβ peptide (Aβ_11–42_), thereby predisposing to AD constituting 5% of total cases [31, 32]. Transmembrane APP protein is synthesized by ER-localized ribosomes, then, enters the ER lumen for posttranslational modification to correct mistakes in folding [33]. The *APP* expression predominantly occurs in astrocytes, oligodendrocytes, and neurons in the brain [34].

Neurons have acquired a complex network of transcriptional effectors and sensors to sustain healthy protein homeostasis [35]. During aging, there is a gradual decline and perturbation of protein homeostasis via excessive accumulation of aberrantly ubiquitinated, oxidized, or misfolded proteins in neurons [36, 37]. Upon accumulation of such proteins in the ER lumen, the UPR response is commenced to either reinstate protein homeostasis or ignite cell death upon irreversible stress [38, 39]. Accumulating evidence suggests that the progressive accumulation/aggregation of hyperphosphorylated tau protein or Aβ peptides in AD induces irreversible ER stress, thereby, causing synapse dysfunction and neurodegeneration [40].

In summary, AD is associated with the accumulation of unfolded proteins, metabolic derangements, and enhanced oxidative stress in the diseased neurons, as well as with neuroinflammation involving also glial cells, such as microglia and astrocytes adjacent to these unhealthy neurons. Under these circumstances, the ER homeostasis is dampened. As a result, the AD brain manifests ER stress [41, 42]. Herein, we will elucidate the participation of ER stress in the pathogenesis of AD in an attempt to reveal possible novel strategies, therapeutics, and molecular targets for the intervention and management of AD pathology.

## ER stress and unfolded protein response (UPR): Adaptive versus maladaptive

**Fig. 1:**
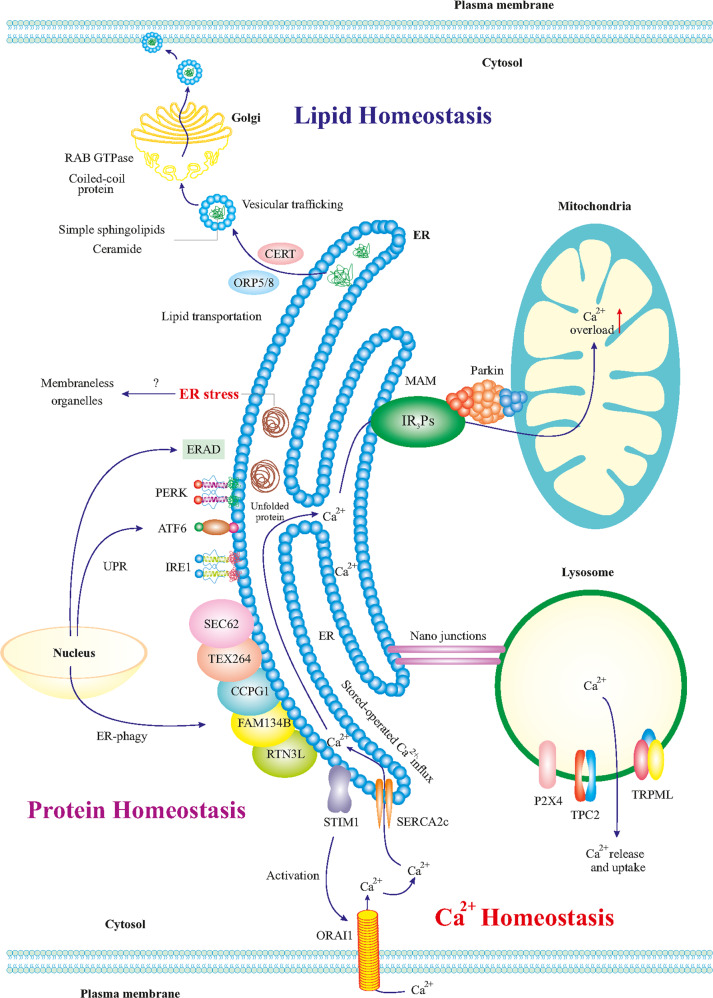
The ER and lipid, protein, and Ca^2+^ homeostasis in neurons. The ER is in direct or indirect communication with other organelles such as mitochondria, other organelles, plasma membrane, phagosomes, lysosomes, and endosomes. Such communications regulate the metabolism and homeostasis of lipids, proteins, and Ca^2+^. ER-produced lipids including sphingolipids and ceramide are trafficked to the Golgi via vesicles, mediated by CERT and ORP5/8 proteins. In Golgi, RAB GTPases and coiled-coil proteins mediate the final trafficking of the lipids to the plasma membrane. ER stress can influence mitochondria functions and vice versa and the contact points mitochondrial associated membranes (MAM) contain crucial proteins for neuronal physiology. For example, Ca^2+^ transportation to the mitochondria is influenced by MAM, which may lead to mitochondrial Ca^2+^ overload, reduced energy capacity, and oxidative stress in the neuron. Besides, nano-junctions between the ER and lysosomes modulate Ca^2+^ signaling in the ER. ER-mediated activation of the ORAI1 transporter leads to the influx of intracellular Ca^2+^ to the ER. In addition, it is thought that ER stress is affected by ER communication with membrane-less organelles. The roles of the UPR and ER-phagy (autophagy of the ER) for the turnover of the different components in cell homeostasis and their dysregulation in disease conditions such as AD is currently receiving more attention.

**Fig. 2:**
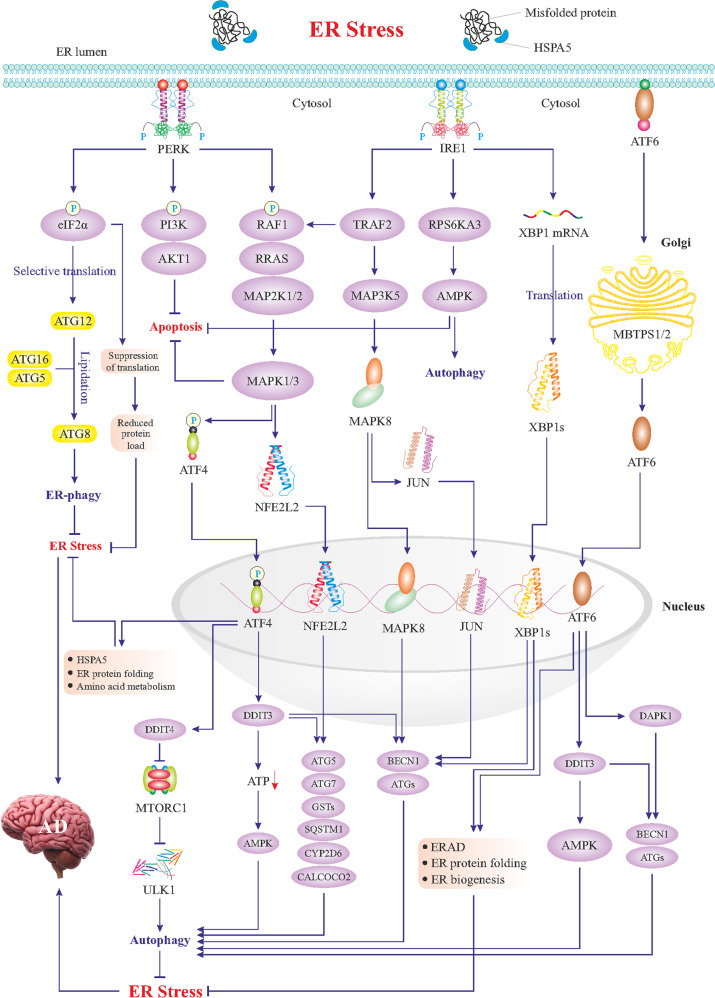
Mild ER stress and adaptive UPR signaling. Mild ER stress triggers adaptive UPR signaling composed of three main branches; PERK, IRE1, and ATF6. As shown in the figure, the PERK–eIF2α axis promotes selective translation of some genes such as ATG12, which along with ATG16 and ATG5 induces lipidation and activation of ATG8, resulting in autophagy of the ER (ER-phagy). PERK-mediated phosphorylation of eIF2α also suppresses the translation to reduce protein overload in the ER. Likewise, the PERK–PI3K–AKT1 axis blocks apoptosis, and the PERK–RAF1–RRAS–MAP kinases cascade activates two cardinal transcription factors, ATF4 and NFE2L2, which transactivate genes encoding proteins involved in autophagy. mTOR complex 1 (mTORC1) is a negative regulator of autophagy via suppression of the ULK1 signaling complex to beclin1(BECN1). Inhibition of mTORC1 by DDIT4 can in turn activate autophagy. NFE2L2-induced genes encode components of autophagy machinery further fueling autophagy. NFE2L2 also upregulates antioxidant genes including CYP2D6 and CALCOCO2. Among ATF4-upregulated genes are those encoding ER chaperones such as HSPA5 and other enzymes to facilitate protein folding in the ER. Activation of the IRE1 branch during the UPR leads to the activation of some key transcription factors (see figure). Thus IRE–TRAF2 axis can activate i MAPK8 and JUN, which relocate to the nucleus and upregulate *ATGs* and *BECN1* genes. IRE1-mediated activation of the AMPK also boosts autophagy and blocks apoptosis. Most importantly, IRE1 via its inherent RNAse activity produces mRNA encoding the transcription factor XBP1s, which in the nucleus upregulates autophagy-associated genes and proteins involved in the ERAD. During the UPR, ATF6 is processed in the Golgi to produce the active transcription factor which in turn can also upregulate genes encoding chaperones and ERAD proteins as well as *DAPK1* and *DDIT3 genes*, with a role in autophagy. Overall, the adaptive UPR suppresses ER stress via induction of corrective autophagy, inhibition of apoptosis, and activation of the ERAD. There is also an upregulation of ER chaperones, inhibition of additional protein translation, and an enhancement of ER capacity all serving to boost the correct folding of proteins.

**Fig. 3:**
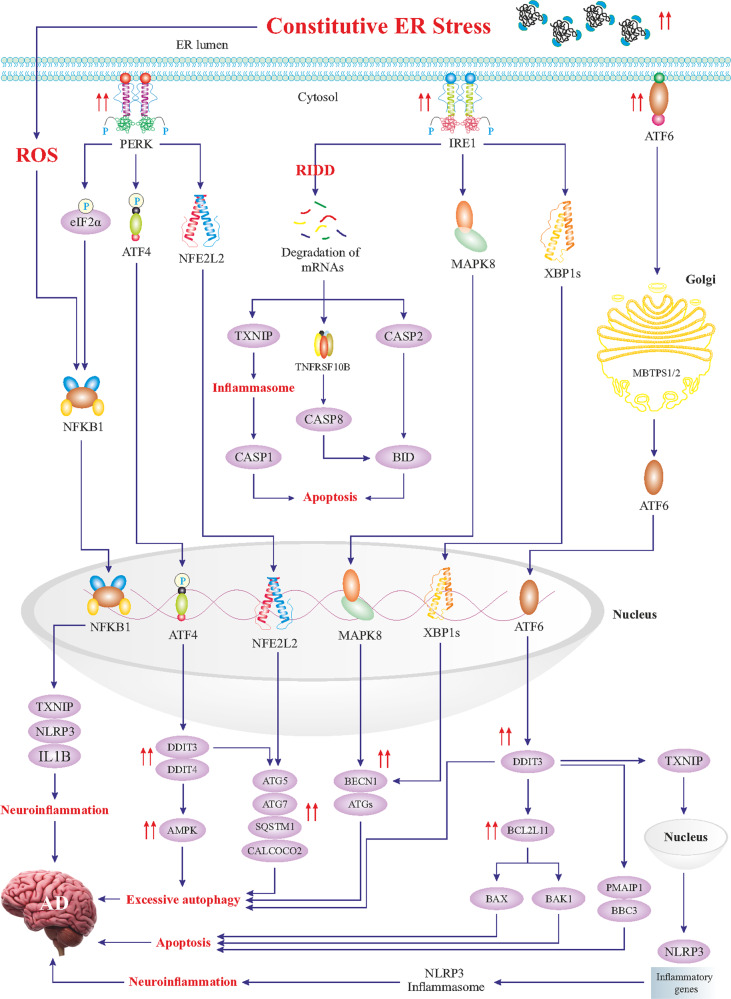
Constitutive ER stress and maladaptive UPR signaling. Constitutive ER stress triggers maladaptive UPR signaling characterized by excessive activation of the UPR branches. Hyperactivated PERK signaling leads to the activation of NFKB1, igniting neuroinflammation. Prolonged phosphorylation of eIF2alpha causes a block in the synthesis of crucial synaptic and other proteins necessary for neuronal functions. In addition, hyperactivated ATF4 results in an excessive upregulation of *DDIT3* and *DDIT4* genes with an enhanced expression of autophagy genes, which is detrimental to neurons. Likewise, NFE2L2-induced hyper-transactivation of autophagy genes will lead to excessive autophagy. Overactive IRE1 will lead to massive degradation of mRNAs, being referred to as the RIDD (regulated Ire1-dependent decay), and subsequent induction of apoptosis and neuroinflammation. MAPK8 contributes to excessive autophagy via an enhanced upregulation of autophagy genes and ATF6- and ATF4-transactivated *DDIT3* upregulates pro-apoptotic genes, leading to neuronal cell death. DDIT3-induced upregulation of the *TXNIP* gene, encoding a transcription factor, upregulates neuroinflammatory genes, and promotes the formation of the NLRP3 inflammasome, leading to neuroinflammation. Overall, a maladaptive UPR is characterized by excessive autophagy, apoptosis, and severe neuroinflammation, worsening the pathology observed in AD.

The ER is a relatively large eukaryotic organelle forming a network of membranous and flattened sacs extending across the cytoplasm and abutting the nucleus (Fig. 1) [43]. ER hosts cardinal cellular processes including protein biosynthesis, folding, modification, and assembly (Fig. 1) [44]. The ER-resident ribosomes synthesize nearly one-third of the total cellular proteins, which translocate to the ER lumen to acquire proper folding and three-dimensional structures prior to transport to target organelles and plasma membrane [45]. Moreover, the ER stores intracellular Ca^2+^ dynamically in response to changes in redox balance, nutrients, energy, hormones, and growth factors (Fig. 1) [46]. Under certain physiological or pathological states, the demand for protein biosynthesis rises dramatically, exceeding the protein-folding capacity of the ER lumen, culminating in the formation of partially folded, misfolded, or unfolded proteins—a state commonly known as ER stress [12, 47, 48]. To resolve ER stress, the UPR evolves to ensure the folding of proteins, and ward off the overwhelming accumulation of misfolded/unfolded proteins in the ER lumen [49–51]. The UPR commences a whole range of signaling cascades which modifies cellular transcriptional and translational events in an attempt to cope with ER stress and reinstate the ER homeostasis [52–54]. Typically, mild ER stress can be resolved by the UPR, termed “adaptive or cytoprotective UPR”. Excessive, prolonged, or constitutive ER stress, however, induces prolonged activation of the UPR, termed “maladaptive or terminal UPR”, leading to the induction of cell death pathways (Figs. 2 and 3) [12, 47]. In terms of mechanism of action, adaptive UPR and maladaptive UPR share similar signaling patterns in the cellular events within the realm of ER stress. Nonetheless, it is perceived that the major difference between adaptive and maladaptive UPR resides in the level of ER stress and the corresponding magnitude/duration of the UPR activation [12, 47].

UPR signaling constitutes three key ER stress sensors in the ER membrane encompassing protein kinase R-like endoplasmic reticulum kinase (PERK, encoded by *EIF2AK3* gene), inositol-requiring enzyme 1 (IRE1, encoded by *ERN1* gene), and activating transcription factor 6 (ATF6) [55–57]. Under a physiological state, these sensors remain inactive by binding to a cluster of ER-localized HSPA5 chaperones (aka BiP or GRP78) [58]. Upon ER stress challenge, HSPA5 chaperones bind to misfolded/unfolded proteins, thus releasing PERK, IRE1, and ATF6, to sense unfolded/misfolded proteins with their ER-luminal domains and transmit signals through cytosolic domains (Figs. 2 and 3) [59]. PERK undergoes homodimerization and *trans*-phosphorylation, thereby, phosphorylates eukaryotic translation-initiation factor 2α (eIF2α, encoded by *EIF2A* gene) on the α-subunit (Figs. 2 and 3) [60]. The phosphorylated eIF2α perturbs the *80S ribosome* assembly inhibiting protein translation, thus, and blocking the production of the additional influx of nascent polypeptides that could worsen the ER stress. In contrast, phosphorylated eIF2α mediates the translation of certain proteins, such as the ATF4 transcription factor that translocates to the nucleus and transactivates UPR target genes, encoding proteins and alleviating ER stress. However, during constitutive ER stress activation of the PERK can mediate cell degeneration by induction of specific cell death genes (Figs. 2 and 3) [12, 47, 61, 62]. IRE1 exhibits a protein-kinase activity, leading to IRE1 autophosphorylation and consequently, activation of its endoribonuclease activity [63]. Subsequently, IRE1 cleaves a 26-base intron from *XBP1* mRNA, resulting in the translation of a spliced XBP1 protein (XBP1s), which functions as a transcription factor to upregulate UPR target genes [64]. Likewise, hyperactivation of IRE1 is perceived to induce overexpression of UPR target genes, leading to maladaptive response and cell death (Fig. 3). ATF6 is also a transcription factor belonging to the ATF family, capable of relocating to the Golgi apparatus to yield its active form through MBTPS1- and MBTPS2-mediated cleavage (Fig. 2) [65, 66]. Cleaved and activated ATF6 relocates to the nucleus to transactivate genes encoding chaperones and proteins implicated in the folding, maturation, and secretion of proteins, as well as ER-associated protein degradation (ERAD), a process by which ER-trapped misfolded/unfolded proteins are transferred to the cytosol for degradation by *proteasomes* [67, 68]. As aforementioned, if proper UPR fails to restore ER homeostasis, it may develop into maladaptive UPR machinery igniting cell death, mainly apoptosis [53]. ER stress-induced apoptosis is mainly driven by the DDIT3 transcription factor, which transactivates multiple apoptotic genes (Fig. 3). Other than these branches of UPR, hyperactivation of *autophagy* may also participate in the induction of cell death upon excessive ER stress (Fig. 3).

## ER stress and the UPR in Alzheimer’s disease

### Aβ peptides deposition and neuronal ER stress in AD

*APP* and *PSEN1* genes encode transmembrane proteins with one (APP) and nine domains (presenilin 1), respectively [69, 70]. Both of these proteins participate in the formation of Aβ peptides and their mutations and mutation of the related *PSEN2* are observed in patients with early onset or familiar AD. Overexpression of *APP* and *PSEN1* was shown to induce a misfolded configuration of these proteins in the ER, causing ER stress [71]. Meanwhile, it is perceived that Aβ peptides accumulation, which is closely correlated with high expression levels of *APP* and *PSEN1*, triggers neuronal ER stress during the early stages of AD [22]. To support this notion, *Soejima* and associates examined the aggregation of Aβ oligomers, toxic turn Aβ (at positions 22 and 23), and Aβ in brains of AD patients and triple transgenic (3 × Tg)-AD mice, as well as in SH-SY5Y cells transfected with *PSEN1* gene (participates in the formation of Aβ) [72]. Their findings revealed that protein levels of RAB4A, RAB6A, and HSPA5 were upregulated in *PSEN1* transfected SH-SY5Y cells and accumulated in 3 × Tg-AD mice neurons [72]. HSPA5 is an ER stress marker, upregulation of which is indicative of ER stress induction owing to the accumulation of toxic turn Aβ [72]. Therefore, these findings indicate that intraneuronal aggregation of toxic turn Aβ triggers ER stress in the early stage of AD in both murine models and human AD. However, this study did not distinguish the adaptive or maladaptive characteristics of the UPR and the degree of ER stress induction in neurons in the early stage of AD. We hypothesize that Aβ deposition in the ER triggers mild ER stress in the early stage of AD, resulting in adaptive UPR in an effort to revert ER stress. However, it is plausible to speculate that the constitutive and excess ER stress during the advanced stage of AD leads to maladaptive UPR, neuronal cell death, and progression of AD. Along the same line, work carried out by Lee et al. has shown that deposition of Aβ in human SK-N-SH neuroblastoma cells could induce both adaptive, through activation of HSPA5, and maladaptive UPR pathways with an increase in pro-apoptotic factors such as DDIT3 and CASP4 (Figs. 2 and 3) [73]. Silencing *PERK* promoted Aβ neurotoxicity due to inhibition of adaptive UPR in neurons [73]. On the other hand, treatment with salubrinal (see the section “Salubrinal”), a positive regulator of eIF2α, markedly upregulated HSPA5 chaperone, and hampered CASP4 activation, ultimately, resolved ER stress in neurons [73]. These findings point to an adaptive role of the PERK-eIF2α signaling in the alleviation of ER stress, induced by Aβ deposition in AD. Salubrinal could be a plausible therapeutic agent to restore ER homeostasis and ameliorate AD and Aβ pathology. This study further corroborates the view that Aβ deposition initially triggers mild ER stress, and adaptive UPR to reinstate ER homeostasis. Besides the PERK branch, *Aniotz* and their team unraveled that activation of the IRE1 branch of UPR is correlated with human AD pathology [74]. Genetic ablation of the endoribonuclease domain of IRE1 depleted Aβ deposition and oligomerization, improved memory, learning capacity, long-term potentiation, and enhanced synaptic capacity in AD mice [74]. Moreover, *ERN1* ablation overtly downregulated APP levels in the hippocampus and cortical areas in AD mice [74]. In vitro experiments showed that inhibition of IRE1 downstream signaling also decreased steady-state levels of APP, causing its confinement within the ER to ensure its degradation driven by proteasomes [74]. Collectively, these findings reveal a pathological role of IRE1 signaling of UPR in AD. The rationale regarding the involvement of the IRE1 branch of UPR may be justified by its participation in the maladaptive UPR domain. Although the IRE1 branch of UPR commences adaptive signaling machinery under mild ER stress, constitutive activation of the IRE1 branch is perceived to be detrimental due to the overwhelmed induction of pro-apoptotic and pro-inflammatory signaling in neurons (Fig. 3). Hence, it can be postulated that both PERK and IRE1 signalings share downstream pro-inflammatory or pro-apoptotic pathways upon excessive ER stress, resulting in neuroinflammation or cell apoptosis.

Another important feature of AD pathology is the involvement of the neurovascular unit which is composed of neurons, astrocytes, endothelial cells of the blood–brain barrier, and pericytes. Data from the literature showed that impairment of brain endothelial cells causes neurovascular unit dysfunctions, likely contributing to the pathogenesis of AD [75]. *Fonseca* and associates examined the hypothesis that Aβ deposition in cerebral vessels ignites constitutive ER stress and pro-apoptotic UPR [76]. They incubated rat RBE4 cell line with Aβ_1–40_ (the most abundant isoform of Aβ in the brain) and discerned upregulation of ER stress markers in a time-dependent fashion [76]. Aβ_1–40_ accumulation triggered neuronal cell death contingent on mitochondria and caspase activation, demonstrated by CASP9 and 12 activations, as well as elevated cytochrome c release from mitochondria [76]. Also, Aβ_1–40_ accumulation fostered nuclear translocation of the AIFM1 apoptotic factor [76]. Finally, these authors unveiled that constitutive ER stress induced by Aβ_1–40_ underpinned neuronal death via maladaptive UPR pathways [76]. These results lend credence to the notion that Aβ_1–40_-induced constitutive ER stress contributes to the death of brain endothelial cells. In view of this idea, containment of ER stress could be a therapeutic scheme to reinstate neurovascular unit function in AD. Taken together, the data presented suggest that Aβ deposition is linked to induction of ER stress and upregulation/activation of HSPA5 and PERK-eIF2α signaling to resolve ER stress and regain ER homeostasis. However, as AD progresses maladaptive UPR is activated through upregulation of *DDIT3*, *CASP4*, and other pro-apoptotic pathways, leading to neuronal death.

Concomitant with these findings, abundant in vivo evidence suggests that AD is associated with induction of constitutive ER stress and maladaptive UPR, which contribute to AD pathology. Cui and associates revealed that *PTEN* and ER stress markers (*HSPA5* and *DDIT3*) were upregulated in AD transgenic (*APP*/*PSEN1*) mice [77]. Generally speaking, PTEN plays a crucial role in the regulation of neuronal survival and differentiation [78]. However, findings of this study showed that pharmacological inhibition of PTEN suppressed ER stress, apoptosis, and induced phosphorylation/activation of PIK3CA (PI3K)/AKT1 (a crucial axis regulating cell division, survival, and growth, and negative regulator of apoptosis) (Fig. 2), leading to suppression of apoptosis in the hippocampus and amelioration of AD phenotype [77]. These observations suggest that AD pathology is linked to constitutive induction of ER stress and maladaptive UPR, leading to neuronal loss through ER stress-induced apoptosis (Fig. 3) [77]. Overall, these findings suggest that maladaptive UPR observed in a murine model of AD is accompanied by upregulation of not only *HSPA5* and *DDIT3* but also *PTEN*, indicating a link between PTEN and other components of maladaptive UPR. Hence, inhibition of PTEN is a potential strategy to reconcile neuronal apoptosis induced by maladaptive UPR in AD. In addition, calpains are a superfamily of cysteine proteases, which are ubiquitously expressed in human cells and mediate the degradation of intracellular proteins, with an established role in cellular processes such as apoptosis and cellular proliferation [79]. In a recent study, Wang and colleagues examined the notion that hypoxia-mediated activation of CAPN2 (m-calpain) is associated with ER stress and AD pathogenesis [80]. They found that hypoxia compromised memory and spatial learning in *APP*/*PSEN1* transgenic mice [80]. Moreover, hypoxia-upregulated *CAPN2*, induced tau hyperphosphorylation, Aβ deposition, and ER stress, resulting in apoptosis in *CAPN2* transgenic mouse brain, indicating activated maladaptive UPR [80]. Silencing the *CAPN2* gene in SH-SY5Y cells reversed hypoxia-induced effects and suppressed ER stress, apoptosis, and tau hyperphosphorylation [80]. These findings support that hypoxia-induced upregulation of *CAPN2* gene/protein leads to neuronal cell death through maladaptive UPR. As a consequence, CAPN2 could be a potential target to consider in the management of AD in the context of maladaptive UPR. Overall, besides the major mechanisms of maladaptive UPR, which are initiated by UPR branches and their signaling cascades (Fig. 3), PTEN and calpains are suggested to be secondary effectors of maladaptive UPR in AD [80].

RAB6A is a small GTPase from the RAB6 superfamily, with a role in ER–Golgi trafficking and post-ER quality control [81]. *Scheper* and coworkers evaluated the expression of RAB6A in several brain areas including the temporal cortex, entorhinal, and hippocampus of AD patients [82]. They found that RAB6A was upregulated in all areas tested through an ER stress and post-ER quality control-dependent mechanism [82]. Concomitantly, *Elfrink* and colleagues reported that RAB6A levels were increased proportionally to the extent of ER stress and UPR activation in the brains of AD patients [83]. The functional role of RAB6A was believed to counteract maladaptive UPR and constitutive ER stress during the early stage of AD [83]. These data suggest that RAB6A confers protection against maladaptive UPR and therefore, holds therapeutic promises in reverting ER stress in AD. Nonetheless, more studies are necessary to unveil the underlying mechanism of RAB-mediated protection against maladaptive UPR.

At variance with the abovementioned observations, there are also studies suggesting that AD and Aβ pathology are not related to ER stress and the UPR. *Sadleir* and colleagues examined the 5XFAD mice, a common model of AD overexpressing *PSEN1* and *APP* genes [84], and showed that they do not manifest an upregulation of ER stress markers (e.g., HSPA5, IRE1, DDIT3, ATF4, and eIF2α) [84]. Hence, these data suggest that higher *APP* and *PSEN1* expression and AD pathology might be independent of ER stress or UPR activation in the 5XFAD mouse model of AD [84]. Furthermore, *Hashimoto* and his team utilized an *App*-knock-in mouse model of AD, and displayed Aβ accumulation independent of *APP* overexpression [85]. Their findings did not favor an ER stress induction in *App*-knock-in or single *App*-transgenic mice [85]. In summary, despite the majority of evidence obtained from AD mouse models in vivo [72, 74, 76, 77, 80] supporting the role of excessive ER stress and maladaptive UPR in the pathogenesis of AD, a few studies [84, 85] do not. One possible explanation for this discrepancy observed in various AD mouse lines may be related to the different cell types and brain areas analyzed, the timing of the measurement and the techniques employed. As discussed in this review, the different concepts of ER stress, adaptive UPR vs. maladaptive UPR could be helpful to understand better the pathophysiological responses occurring in vulnerable neurons in AD. Finally, the implementation and analysis of human cells and brain samples (postmortem and others) from AD patients could be an experimental approach in the future to resolve these conflicting observations.

### Mild ER stress and adaptive UPR signaling in AD

In eukaryote cells, there are two major systems for protein degradation, the ubiquitin–proteasome system (UPS) [86] and autophagy [16]. Both these systems take part in normal protein turnover in cells and are also involved in the disposal of unfolded/misfolded proteins accumulating in various diseases. There is also crosstalk between UPS and autophagy that can involve key regulators such as the deubiquitinating enzyme Usp14 [87]. Dysfunctional UPS and autophagy regulation is observed in many human conditions including neurodegenerative diseases [38]. Nijholt and colleagues explored the impact of UPR on proteolytic capacity in a murine model of AD and showed that *PSMB10* and *PSMB8* genes encoding immunoproteasome subunits were upregulated in the brain [88]. Immunoproteasome is a proteasome highly expressed in immune and nonimmune cells and degrades intracellular ubiquitin-labeled proteins, particularly, following inflammation and oxidative stress [89]. Interestingly, the authors observed that the UPR activation was not associated with increased proteasome but upregulated autophagy as a major degradation process in murine AD brain [88]. This suggests that mild ER stress-inducing adaptive UPR signaling can upregulate autophagy but not proteasome as a cytoprotective mechanism in neuronal cells [88]. In contrast, excessive ER stress can trigger maladaptive UPR activation with enhanced autophagy and induction of cell death pathways (Fig. 3) [90]. The function of autophagy can be both disease-promoting and suppressive depending on the context [91–94]. Therefore, the study of the intricate links between autophagy and protein turnover at different stages of AD will be an important avenue to pursue in the future. Overall, the data suggest that mild ER stress/adaptive UPR commonly noted during the early stages of AD is accompanied by activation of mild/adaptive autophagy (Fig. 2). However, excessive ER stress/maladaptive UPR can trigger constitutive/excessive autophagy, which may lead to neuronal death and exacerbation of AD (Fig. 3) [12].

On the other hand, microRNAs (miR) are non-coding RNAs participating in post-transcriptional gene expression regulation through binding to their target mRNAs and inhibiting translation [95]. Wu and colleagues unraveled that *miR-200c* blocked the translation of *PTEN* mRNA, leading to the differentiation and survival of cultured neurons [96]. They further found that neuronal deposition of Aβ provoked ER stress and induced overexpression of *miR-200c* in a transgenic murine model of AD [96]. Pharmacological suppression of ER stress blocked *miR-200c* expression and impaired neuronal survival following Aβ deposition [96]. This study shows that the *miR-200c*-PTEN axis plays a role in response to Aβ deposition in AD and is linked to adaptive UPR. Consistently, as described above [77], PTEN participates in maladaptive UPR and ignition of apoptosis. Therefore, *miR-200c*-mediated inhibition of PTEN serves as an adaptive response during Aβ deposition and ER stress in AD. Also, given the complexity of miRs and their targets in cell physiology, it would be worthwhile to study which other miRs than *miR-200c* can be regulated by ER stress in neurons and in models of AD. As adaptive UPR activation is perceived to ameliorate AD pathology, there is growing evidence suggesting that AD pathology may be attributed, at least in part, to defective adaptive UPR under ER stress. In a recent study, Katayama and colleagues revealed that *PSEN1*/*PSEN2* null and dominant-negative *PSEN1* mutants did not influence UPR activation in mice [97]. However, they demonstrated that *PSEN1* mutants linked to familial AD perturbed adaptive UPR through inactivation of UPR branches including PERK, IRE1, and ATF6, resulting in the progression of AD due to the inability of neurons to cope with ER stress [97]. In sum, these findings suggest that *PSEN1* mutations in familial AD dampen adaptive UPR, leading to prolongation of ER stress and exacerbation of AD pathogenesis.

### Redox state, neuroinflammation, and ER stress in AD

Mota and colleagues explored early events in the pathogenesis of AD in human peripheral blood cells and the transgenic murine model of AD (3 × Tg-AD) [98]. They revealed that oxidative stress, NFE2L2 phosphorylation, and ER stress markers, were all upregulated in peripheral blood mononuclear cells (PBMCs) from mild AD patients and murine transgenic model of AD [98]. In addition, ER Ca^2+^ homeostasis was impaired in these cells [98]. NFE2L2 is a transcription factor that upregulates anti-oxidant genes to confer resistance against oxidative stress (Fig. 2) [99]. Therefore, increased phosphorylation and nuclear levels of NFE2L2 in the murine brain cortex were indicative of early resistance against oxidative stress in AD [98]. Moreover, the *SOD1* gene (encoding an anti-oxidant protein) was downregulated in both murine and human PBMCs [98]. Taken together, these findings denote that oxidative stress is accompanied by NFE2L2 activation early on but unable to modulate its targets, thereby, resulting in loss of SOD1 upregulation and oxidative stress-induced ER stress in the early stages of AD. They support the idea of alleviating oxidative stress and ER stress in the management of AD onset and progression.

Both clinical and experimental evidence has indicated that neuroinflammation contributes to AD pathogenesis (Fig. 3) [100]. Likewise, it is hypothesized that ER stress activates TXNIP protein, which regulates a redox regulator protein TXN to foster the NLRP3 inflammatory pathway in the AD hippocampus [101]. In this regard, Ismael and coworkers analyzed the postmortem human AD hippocampus for TXNIP-NLRP3 inflammasome activation and ER stress markers [101]. They revealed co-localization of TXNIP in microglia and neurons, and upregulation of transcript and protein levels of TXNIP in close proximity to Aβ deposition in the hippocampus of AD patients [101]. Furthermore, ER stress markers (e.g., *DDIT3*, *EIF2A*), *CASP1*, *IL1B*, and *PYCARD* (encoding an effector of NLRP3 inflammasome) were also upregulated in AD hippocampus [101]. These findings suggest that constitutive ER stress in the hippocampus provokes TXNIP-NLRP3 inflammasome, thereby, igniting neuroinflammation (Fig. 3). Therefore, upon constitutive ER stress and excessive activation of major UPR signalings, the secondary signaling pathways (e.g., NFKB1) are activated, ultimately, leading to neuroinflammation through NLRP3 inflammasome activation (Fig. 3). Hence, mitigation of ER stress or inhibiting TXNIP could be a potential therapeutic strategy to ameliorate AD-associated neuroinflammation.

### Tau pathology and ER stress in AD

The *MAPT* gene encodes a microtubule-associated protein called tau that when hyperphosphorylated tends to form fibrils that aggregate and generate insoluble NFTs inside the cytoplasm of neurons in AD and related tauopathies [102]. Several pieces of evidence have revealed a link between pathological tau and ER stress. *Ho* and coworkers revealed that phosphorylated-PERK (p-PERK), p-eIF2α, XBP1s, and DDIT3 were profoundly elevated in the hippocampus region, indicating constitutive ER stress and maladaptive UPR in aged tau transgenic mice (P301L mutant), as well as rat cortical neurons cell culture [103]. Meanwhile, these authors revealed that ER stress-induced hyperphosphorylation of tau protein at Ser396, Ser262, and Thr231 [103]. These findings indicate that p-tau can lead to ER stress, which in turn provokes further hyperphosphorylation of tau and exacerbate AD-like pathogenesis via a vicious cycle and feed-forward reactions. In view of this, therapeutics that may alleviate ER stress and block maladaptive UPR pathways could be utilized to ameliorate tauopathy in AD. Buchanan et al. examined post-mortem AD samples from the lateral temporal cortex and observed increased levels of p-PERK proportional to the pathological tau levels [104]. They further noted that neuroinflammation and ER stress was mostly discernible in the late stage of AD and are correlated with the tau pathology [104]. This study indicates a link between constitutive ER stress and progressive tau pathology, underscoring the importance of these events in AD. In line with this, Hoozemans et al. reported that the chaperon HSPA5 and p-PERK levels were increased in the temporal cortex during the early stage of *neurofibrillary degeneration* [105]. Some in vitro studies also showed a link between ER stress and phosphorylation of tau, where activation of the UPR would result in upregulation of GSK-3β, a major kinase involved in tau phosphorylation [106]. On the other hand, a study investigating the role of UPR activation during the development of tau pathology in AD in vivo showed that UPR activation markers pPERK, pIRE1α, and peIF2α were elevated in AD hippocampus at an early Braak stage of tau pathology. Based on these results, the authors proposed a working model in which activation of the UPR enhances tau phosphorylation and aggregation and precedes NFTs formation in the hippocampus of AD patients [105]. In contrast, in a study using P301S-tau-transgenic mice (a tauopathy mouse model), there was no increase observed in ER stress markers at different ages, suggesting that tauopathy in AD could be independent of ER stress [85]. Overall, a growing number of in vitro and in vivo studies [103–106] suggest that phosphorylated tau is accompanied by upregulation of DDIT3, p-eIF2α, p-PERK, and XBP1s, thus, contributing to maladaptive UPR activation, creating a vicious cycle and promoting enhanced phosphorylation of tau mainly through GSK-3β upregulation. However, conflicting data also exist [85] refuting the implication of ER stress and the UPR in tauopathy of AD. As described above for Aβ, a possible explanation for this controversy in different mouse AD lines might be related to cell types analyzed, brain areas, and timing of the investigation, as well as methods used. More studies are therefore required regarding the links between pathological changes in tau protein and ER stress using cell cultures and in mouse and preferably human models of AD and related tauopathies.

### Vicious cycle between ER stress and insulin resistance in AD

A growing body of evidence suggests that type 2 diabetes predisposes to vascular dementia and stroke thereby, increasing the risk of developing AD later in life [107]. Antidiabetic drugs such as thiazolidinediones, metformin, and agents targeting the glucagon-like peptide-1 receptor have been shown to modulate brain regeneration, neuroinflammation, and metabolism [108]. AD is characterized by alterations in response to insulin and insulin-like growth factor (IGF) and these may exacerbate the progression of the disease [109]. The underlying mechanisms of brain resistance to insulin/IGF in AD are not fully understood, but some authors have hypothesized that the production of ceramide and constitutive ER stress is linked to brain insulin resistance (IR) and the progression of AD [110]. In peripheral organs, it is known that IR leads to dysregulated lipid metabolism, accumulation of ceramide, enhanced inflammation, and ER stress [111]. In line with these concepts, a study showed increased levels of pro-ceramide, ceramide, ER stress, and pro-apoptotic genes in postmortem AD brain tissues, and these changes were correlated with the severity of the disease [110]. These findings support the notion that brain resistance to insulin/IGF in AD can provoke ER stress and maladaptive UPR with induction of pro-apoptotic genes, likely through ceramide accumulation [110]. Altogether these studies also indicate that targeting the vicious cycle of ER stress and insulin resistance could therefore be of importance in coping with dysfunctional cell stress signaling and its consequences in AD pathophysiology.

## Emerging natural/pharmaceutical therapeutics to modulate ER stress and the UPR

**Fig. 4:**
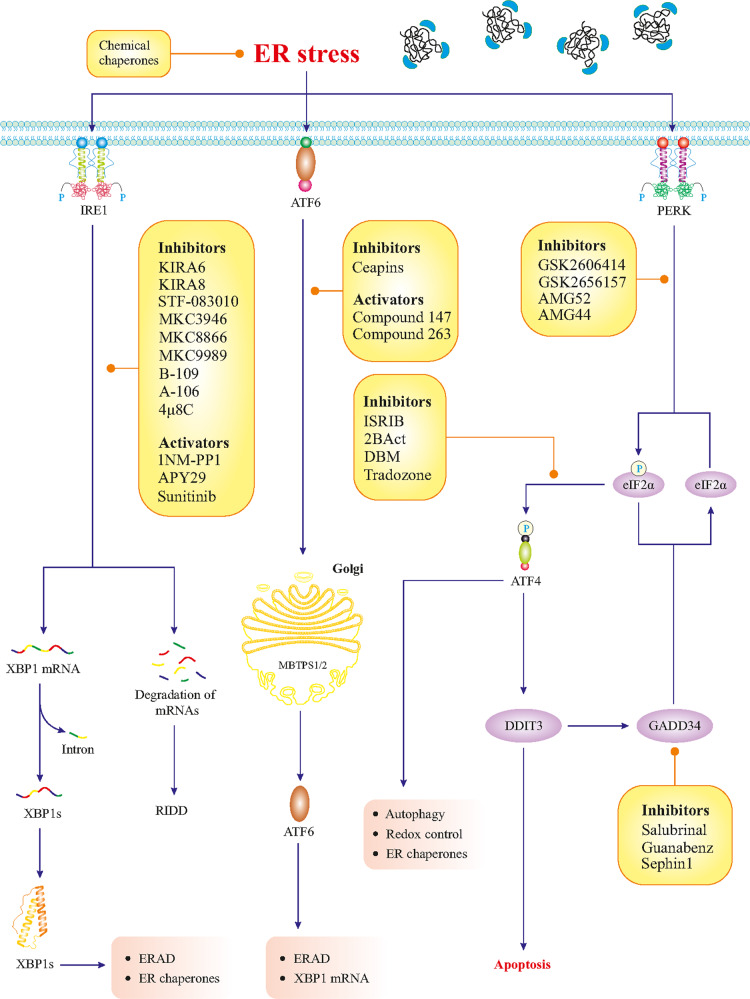
Pharmacological targeting of the UPR. The UPR branches can be targeted and modulated by small pharmaceutical compounds. The list presented here is based on the currently available literature.

Given the implication of maladaptive UPR and ER stress in the pathology of AD, both could be therapeutic targets for future treatments. Compounds derived from natural sources with the capacity to alleviate ER stress may serve as potential therapeutics for maladaptive UPR in AD. Several pre-clinical experiments using cellular and animal models of AD discussed in this review support this view although more detailed studies also in vivo are warranted. An overview of ongoing clinical trials on AD using different compounds can be found in the database, clinicaltrials.gov. Below, we thus summarize some emerging natural/pharmaceutical therapeutics that can target ER stress and the UPR and could be of value also in the management of AD. Figure 4 summarizes several small pharmacological drugs that can target UPR branches [112].

### Berberine

Berberine is a natural isoquinoline alkaloid with specific biochemical and pharmacological characteristics and it has been used in traditional Chinese medicine for over a thousand years [113]. Xuan et al. investigated the therapeutic capacity of berberine in a murine model of combined type 2 diabetes and AD [114]. The study showed that berberine alleviated memory deficits, neuronal damage, and restored lipid and glucose levels in this model [114]. Furthermore, berberine treatment repressed the transcription of ER stress-associated genes [114]. These results support the notion that berberine affords neuroprotection against ER stress and maladaptive UPR occurring in diabetic AD mice. Likewise using the 3 × Tg AD mouse model, Liang et al. reported that berberine was able to suppress the PERK-eIF2α-BACE1 signaling pathway (a key pathway in Aβ production), thereby reducing the production and deposition of Aβ peptides and mitigating cell death [115]. Taken together these observations underscore the ability of berberine to preserve neurons against maladaptive UPR. However, further clinical and experimental studies are required to corroborate these results using human AD models.

### Crocin

Crocin is a natural carotenoid compound derived from the *gardenia* and *crocus* flowers, showing potential therapeutic effects in the alleviation of various neurological disorders [116]. The neuroprotective action of crocin is related to its anti-inflammatory, antioxidant, and anti-apoptotic activities [116]. Using a rat model of AD, Lin et al. showed that crocin can enhance memory and learning ability, mainly through suppression of ER stress and neuronal cell death, as observed in prefrontal cortical neurons and hippocampal CA1 region of the rats [117]. Although more data are needed, the study suggests that crocin can counteract excessive ER stress with beneficial effects on neuronal functions in the rat AD model.

### Luteolin

Luteolin is a natural flavonoid derived from medicinal herbs, vegetables, and many fruits, and has been used in Chinese traditional medicine. Luteolin has also been shown to have anti-cancer, antioxidant, and anti-inflammatory effects in cells [118]. Kou et al. investigated the impact of luteolin treatment on neuroinflammation and memory dysfunctions observed in the 3 × Tg-AD murine model of AD [119]. Data obtained showed that luteolin was able to mitigate memory deficits, improve spatial learning, and downregulate ER stress (*HSPA5*, *ERN1*), and neuroinflammatory markers (*NOS2*, *PTGS2*, *IL6*, *IL1B*, *TNF*) in a concentration-dependent manner [119]. The target of luteolin action could be both neurons and reactive astrocytes that are increased in number in the AD brain tissue [119]. These observations hopefully can spur further research on luteolin and its potential as a therapeutic compound against ER stress and neuroinflammation in human AD models.

### Bajijiasu

Bajijiasu is a natural compound derived from *Morinda officinalis F. C*., a traditional Chinese herbal medicine [120]. Xu et al. studied the effects of bajijiasu in the *APP*/*PSEN1* mouse model of AD, and observed an improvement in memory and learning, as well as a reduction of ROS levels in the cortex and hippocampus of these mice brains [121]. Bajijiasu also afforded neuroprotection against ER stress accompanied by an upregulation of neurotrophic factors in the brain [121]. These observations unravel the therapeutic potentials of bajijiasu in the management of AD by improving cognition and alleviating ER stress. However, so far studies on the effects of bajijiasu in the context of human cells and AD models are lacking.

### Echinacoside

Echinacoside is a natural compound extracted from *Cistanche tubulosa*, a Chinese herbal medicine, manifesting robust neuroprotective effects in multiple neurodegenerative disorders [122]. Studies by Dai et al. revealed that echinacoside can inhibit PERK phosphorylation by binding to PERK with high affinity, suppressing UPR activation [123]. Moreover, echinacoside diminished cerebral deposition of Aβ peptides by blocking *BACE1* mRNA translation (a key enzyme in Aβ production) and enhanced memory in the 2 × Tg-AD transgenic mouse model [123]. These observations are promising and support the potential of echinacoside as a compound that warrants to be further studies in other models of AD.

### Ginsenoside-Rg1

Ginsenoside-Rg1 is a natural neuroprotective compound derived from ginseng with promising neuroprotective effects on neuronal apoptosis in animal models of depressive-like disorders [124]. Mu and associates scrutinized the neuroprotective effects of ginsenoside-Rg1 in the double AD transgenic rat model and showed that deposition of Aβ plaques was markedly reduced following ginsenoside-Rg1 treatment [125]. Ginsenoside-Rg1 also downregulated the expression of *CASP3*, and thereby, by blocking the pro-apoptotic signaling mitigated ER stress and UPR-associated apoptosis [125]. These observations support the potential of ginsenoside-Rg1 in managing AD through ER stress modulation.

### Chrysophanol

Chrysophanol (aka Rhei radix et rhizome) is a natural anthraquinone, which has been utilized in traditional Chinese medicine owing to its potent anti-inflammatory and anti-oxidative stress activities [126]. Li et al. investigated chrysophanol in cell and animal models of AD with Aβ_25−35_ depositions and noted beneficial effects of the compound including a reduction of neuronal apoptosis (evidenced by downregulation of CASP3 and 9), downregulation of ER stress markers, and increased cell survival [127]. In addition to neurons, chrysophanol can act on microglial cells to dampen neuroinflammation [126]. Collectively these findings show that chrysophanol is a potential therapeutic in models of AD by reversing ER stress and averting UPR-induced apoptosis. However, similar to other aforementioned compounds, clinical studies on chrysophanol are highly warranted to corroborate the clinical applicability of these compounds in human AD including safety issues, drug tolerability, and brain penetrance.

### Salubrinal

Salubrinal is a chemical compound that acts through the inhibition of eIF2α dephosphorylation (Fig. 4) and has been shown protective effects in different models of acute injuries [128]. Goswami et al. investigated the effects of salubrinal in the context of neuronal degeneration in an AD rat model receiving 5 μg Aβ_1−42_ daily. The study showed that the administration of Salubrinal was able to reverse the Aβ-mediated upregulation of ER-associated proteins including HSPA5, DDIT3, CASP3, and CASP12 [129]. These are promising data and suggest that salubrinal could be a potential compound to consider in models of AD. However, it is important to keep in mind that since the drug can sustain high levels of phosphorylated eIF2α in the cells, for chronic treatment this fact may be a disadvantage for the overall neuronal protein synthesis and functions.

### Taurodeoxycholic acid

Taurodeoxycholic acid (TUDCA) is a chemical chaperone with the potential to reduce ER stress and is widely employed in ER stress-related cell and animal studies [130]. Ochiai and colleagues investigated the impact of ER stress suppression in obese/diabetic mouse brains with Aβ deposition [131]. They administered TUDCA intraperitoneally to *APP* transgenic mice under a high-fat diet (HFD) intake [131]. As a result, ER stress was suppressed while Aβ deposition and insulin resistance were markedly attenuated in brains and peripheral tissues [131]. Hence, the chemical chaperone TUDCA is a potential therapeutic agent that may ameliorate AD progression by diminishing ER stress.

### Risks and challenges of using pharmacological molecules

Without a shadow of a doubt, it is extremely encouraging the notion that all the abovementioned compounds show potential in targeting ER stress in AD pathophysiology. However, as a chronic disorder, AD would typically require long-term treatment and this fact alone raises the possibility that these compounds might have unwanted effects on the immune system, secretory organs, as well as cognitive functions [112]. Therefore, additional studies investigating this aspect for each pharmacological probe are urgently required. Another challenge is the choice of the appropriate time of treatment that would yield the desired UPR inhibition while retarding or completely avoiding potential off-target risks and effects. Generally, there is evidence that long-term treatment in mice is not well-tolerated but high dosing seems to be better tolerated [112]. Besides, given that both adaptive and maladaptive UPR have shared signaling pathways and that their main differences are based on the activation level, targeting some UPR components may disrupt adaptive UPR, which is required for healthy neuronal homeostasis. Therefore, future research needs to shed more light on the underpinning mechanisms and patterns governing adaptivity or maladaptivity of the UPR signaling responses in order to optimize therapeutic interventions and strategies targeting UPR branches and their signaling pathways in AD.

## Non-pharmacological interventions: exercise and caloric restriction

Besides the natural therapeutics and drug compounds, physical activity and caloric restriction are non-pharmacological interventions, which have shown promising results in the alleviation of ER stress in both experimental and clinical studies of Aβ-dependent pathology and AD. Hong and associates examined the protective impact of exercise training on cerebrovascular dysfunction in a murine model of AD (*APP*/*PSEN1*) [132]. They exercised mice on the treadmill and observed downregulation of *APP* and upregulation of *NOS3* and *AKT1* in AD mouse brains [132]. Furthermore, treadmill exercise downregulated ER stress markers (*DDIT3*, *ERN1*, and *EIF2A*) and pro-apoptotic genes (*BAX*, *BCL2*) [132]. In parallel, another paper reported that treadmill exercise prevented memory loss and attenuated Aβ-42 deposition through inhibition of a key enzyme for Aβ production (BACE1) in the hippocampus and/or cortex of a mouse model of AD (*PS2* mutant) [133]. Moreover, treadmill exercise downregulated *HSPA5* mRNA and suppressed activation of UPR branches and ER stress markers such as PERK, ATF6, XBP1s, and eIF2α, as well as DDIT3, CASP3, and CASP12, denoting an alleviation of ER stress and apoptosis [133]. The same mice had a significant reduction of ER stress-driven inflammation (Fig. 3), as evidenced by the lower levels of TNF-α and IL-1A [133]. These findings strongly favor the notion that physical exercises can attenuate AD-associated cerebrovascular dysfunction and AD pathology through machinery contingent upon ER stress alleviation, inhibition of maladaptive UPR, apoptosis, and neuroinflammation. Interestingly, studies in non-AD disease models also demonstrated the beneficial effects of physical exercise on alleviating ER stress. For instance, in obese mice, aerobic exercise was shown to elicit positive responses due to the suppression of ER stress and thereby, the enhancement of insulin signaling [134].

Caloric restriction specifically refers to the restraint of nutrients overconsumption such as fat, sugar, and amino acids. Caloric restriction lowers weight and alleviates maladaptive UPR/ER stress largely through the upregulation of ER chaperones [135]. Patel and coworkers explored short-term caloric restriction in AD-transgenic mice and observed that it markedly attenuated Aβ deposition by 40–55% [136]. These data suggest that caloric restriction is a healthy dietary habit, lowering AD progression through alleviation of ER stress [136]. On the other hand, the consumption of healthy food products including whole grains, apples, avocado, flaxseeds, chia seeds, nuts, legumes, beans, soy, and olive oil, which elevates high-density lipoprotein (HDL) levels may revert constitutive ER stress reminiscent of caloric restriction [137]. Up-to-date, the mechanism underscoring HDL-induced protection against maladaptive UPR is attributed to the enhancement in the mobilization of 24-hydroxycholesterol and subsequently activation of SMO, which triggers *hedgehog signaling*, resulting in the alleviation of ER stress and inhibition of the UPR-induced apoptosis [137]. Also, similar results might be extrapolated to AD models. Altogether, practicing physical activity, caloric restriction, and a daily healthy diet lifestyle may serve as noninvasive means for the alleviation of ER stress and suppression of maladaptive UPR in the management of the age-related risk to develop AD.

## Concluding remarks and future perspectives

Recent evidence has cast new lights on the link between ER stress, UPR, and AD pathogenesis in cellular and animal models as well as human subjects. Although few conflicting studies exist, the majority of the observations have demonstrated the vicious correlation between constitutive ER stress and maladaptive UPR in AD pathogenesis and progression. It is perceived that mild ER stress occurs in the early stage of AD and the activation of the UPR is an adaptive and neuroprotective mechanism. In contrast, excessive and prolonged ER stress occurs predominantly in the advanced stages of AD and is best described as maladaptive UPR that contributes to the worsening of the disease. In this regard, suppressing maladaptive UPR by pharmacological and non-pharmacological means, while maintaining adaptive/basal UPR, can be a useful scheme to consider in the amelioration and management of AD. However, to be efficient and successful several aspects have to be taken into account before therapeutic interventions including timing, safety, drug availability, kinetics, other treatments, and patient-specific conditions. At the early stage of AD, mild ER stress is more dominant, and therefore, the maintenance of basal UPR is imperative to resolve ER stress in neurons. However, with the progression to the advanced stages of the disease, the UPR is highly activated and is characterized by maladaptive responses, therefore, suppression of the UPR could be the right therapeutic approach to consider. While the translation of the results from cell to mouse studies is generally straightforward, this, unfortunately, is not the case for human studies. We conclude that to meet the current clinical needs, the aforementioned UPR-targeting molecules and therapeutics require further development to corroborate their applicability in human AD.

## Supplementary information

checklist

## Data Availability

All the data supporting the findings of this study are available from the corresponding author on reasonable request.
